# Expertise in Musical Improvisation and Creativity: The Mediation of Idea Evaluation

**DOI:** 10.1371/journal.pone.0101568

**Published:** 2014-07-10

**Authors:** Oded M. Kleinmintz, Pavel Goldstein, Naama Mayseless, Donna Abecasis, Simone G. Shamay-Tsoory

**Affiliations:** 1 Department of Psychology, University of Haifa, Haifa, Israel; 2 Department of Statistics, University of Haifa, Haifa, Israel; 3 The Graduate School of Creative Arts Therapies, University of Haifa, Haifa, Israel; Catholic University of Sacro Cuore, Italy

## Abstract

The current study explored the influence of musical expertise, and specifically training in improvisation on creativity, using the framework of the twofold model, according to which creativity involves a process of idea generation and idea evaluation. Based on the hypothesis that a strict evaluation phase may have an inhibiting effect over the generation phase, we predicted that training in improvisation may have a “releasing effect” on the evaluation system, leading to greater creativity. To examine this hypothesis, we compared performance among three groups - musicians trained in improvisation, musicians not trained in improvisation, and non-musicians - on divergent thinking tasks and on their evaluation of creativity. The improvisation group scored higher on fluency and originality compared to the other two groups. Among the musicians, evaluation of creativity mediated how experience in improvisation was related to originality and fluency scores. It is concluded that deliberate practice of improvisation may have a “releasing effect” on creativity.

## Introduction

Creativity plays an important role in the arts, in invention and in innovation, as well as in everyday life [1]. Creativity is generally divided into four domains also known as “The Four P’s”: (1) product, (2) person, (3) process, and (4) press (external forces that influence the other three domains) [2], [3]. Common creativity measures such as divergent thinking tasks usually measure aspects of idea generation. Divergent thinking is a cognitive sub-structure of creative cognition, which reflects the ability to produce multiple answers to a single problem. It is employed in different types of tasks (e.g., Alternative Uses Task [AUT] and Torrance Tests of Creative Thinking [TTCT]), each of which presents a problem and elicits multiple solutions, while measuring the number of items produced (fluency), the statistical rarity of the responses (originality), the number of categories (flexibility), and the number of additional details provided by the participant (elaboration) (see [3] for review). Through evaluation on these general creativity tasks, artists were shown to be highly creative in comparison to non-artists [4]–[8]. Although these findings indicate that training in art may have an effect on improving creativity, no study to date has examined the relationship between specific type of artistic training and creativity among musicians.

Sternberg and Lubart [9] proposed that a creative product must be both novel and appropriate, a distinction that lies at the base of twofold models of creativity. According to the Geneplore model, creativity entails cyclic motion between two phases: *idea generation* and *idea evaluation* [10]–[13]. The idea generation phase entails associatively activating different ideas, connecting these ideas by putting them together in different and unusual ways, and reorganizing these connections in order to create an original product [14]. The idea evaluation phase is in charge of labeling these generated ideas according to their novelty and perceived appropriateness and returning the ideas for additional expansion during the idea generation phase [11], [13].

Several theories have addressed the relationship between the generation and evaluation phases. One possibility is that the evaluation phase regulates generated ideas and may thus inhibit the number of generated ideas (for review see [13]). Thus, some ideas may be rejected during the evaluation phase, in turn limiting the quantity of the creative product.

**Figure 1:**
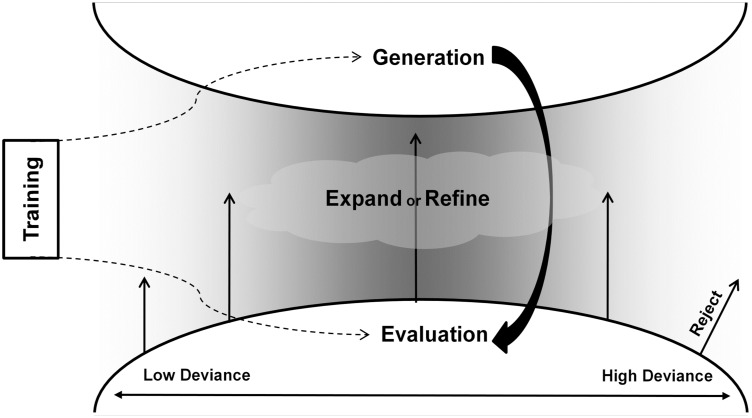
Diagram depicting the suggested twofold model of creativity. The model includes two main units: generation and evaluation. A cyclic motion between generation and evaluation through expansion, focus or rejection of ideas is possible. Medium deviant ideas are more likely to become expanded and focused on, while highly deviant ideas are rejected. Training is a latent variable that can influence both the idea generation phase and the idea evaluation phase.

In line with this notion, in the current study we expanded the twofold model to reflect the suggestion that some ideas are rejected. Moreover, we hypothesize that inhibition is the mechanism involved in the rejection of ideas. According to our model, evaluation is a continuous process taking place along a continuum ranging from stringent to lenient evaluation. Thus, the result of stringent evaluation will not be to expand or focus on an idea, but rather to reject it (see Fig. 1). Several lines of neuropsychological research support the inhibitory mechanism hypothesis. Nijstad et al. [15] found that individuals with reduced latent inhibition (LI) are more creative, leading to the claim that lower inhibition of new ideas may be the source of this advantage. Additionally, it has been suggested that functional as opposed to dysfunctional impulsivity may be associated with higher creativity [16]. Furthermore, neuropsychological evidence suggests that degeneration of anterior, frontal or temporal brain regions may have a “releasing” effect on creativity, pointing to the existence of a system that inhibits creativity [17]–[19]. Collectively, these studies support the notion that the evaluation phase may involve the inhibition of ideas. Thus, according to our model a stringent evaluation phase may be related both to neural and to cognitive inhibition of ideas, eventually leading to lower creativity. Accordingly, the effect of training that involves decreasing the activity of the evaluation system may be to increase creative output.

In line with this view, Fink and Woschnjak [20] examined three groups of dancers and showed that the beneficial influence on creative abilities of certain forms of training, such as *improvisation,* might be greater than the impact of other forms of training (e.g., in ballet). Improvisation is considered to be a progressive form of problem-finding and problem-solving [21], [22]. Training in musical and theatrical improvisation has been found to have an enhancing effect on performance on divergent thinking tasks [23]–[26]. As noted by Thompson specifically with respect to musical improvisation, “…all performances involve some degree of improvisation…” [27], though qualitative differences exist among the creative possibilities in different styles of music. For example, jazz improvisation requires manipulation both of the syntactic or so-called *primary* parameters of music (e.g., melody, harmony, and rhythm; [28]) and of the *secondary* parameters (e.g., loudness, timbre, timing, and tempo). Classical musicians, in contrast, have more restrictions and mainly manipulate the *secondary* parameters of music [27]. While the different emphases in the training of today’s jazz and classical instrumentalists appear to yield a similar level of control over their instruments, these musicians differ in their ability to create something new within the appropriate structure in real time, or in other words, in their ability to improvise [29]. Hence, in line with Ericsson’s [30], [31] concept of “deliberate practice,” according to which specific training and expertise have a substantial influence on the ultimate achievement of creative ability and creative output [30], [31], it may be that it is not only the amount of training that contributes to creativity but also the specific type of training.

**Figure 2:**
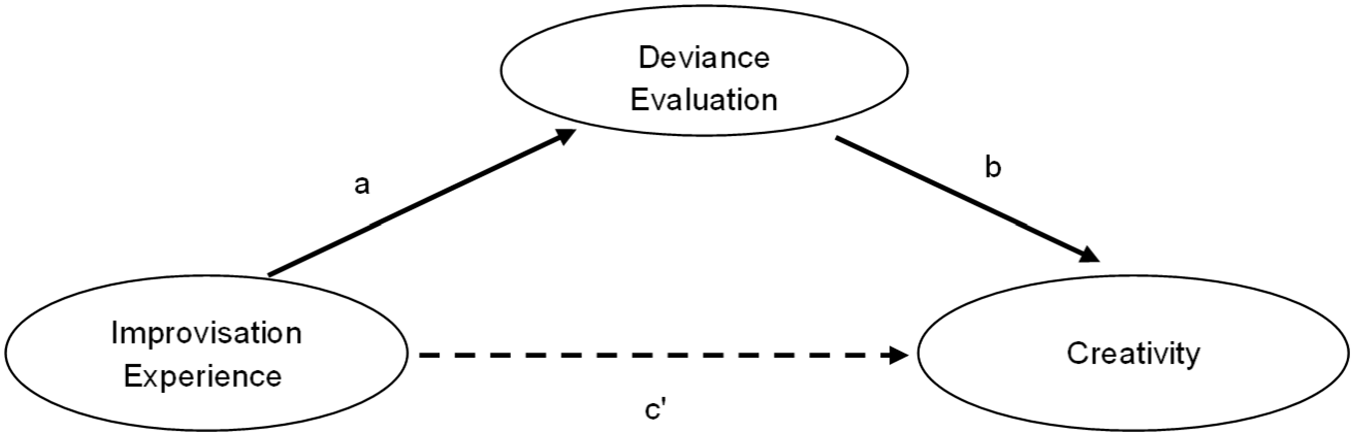
Diagram depicting the indirect effect of improvisation on creativity through the perceived deviance of ideas.

One possible explanation for findings showing greater creativity among musicians is that training and expertise modulate creative output by altering the evaluation phase of creativity. In general, this line of thought considers creativity to be a skill or an ability that can be developed. This view is in line with sociocultural frameworks claiming that the environment (society and culture) can influence people’s evaluation of their creativity and their ability to make creative decisions, thus affecting creative expression [3], [32], [33]. For example, intercultural studies comparing East Asian and Western participants found that the perceived appropriateness of a product, specifically how *deviant* (strange and inappropriate) it is, influences the degree to which the product appeals to specific individuals, resulting in fewer people choosing it in Eastern cultures and more people choosing it in Western cultures [34]–[36]. Furthermore, studies have repeatedly demonstrated that when participants expect external evaluation of their products their creativity scores generally drop (e.g. [37] for review). Additionally, it was argued that external evaluation involves a cyclic motion between one’s personal idea generation and the constraints imposed by the field’s “gatekeepers”, the people who decide what is creative [38]. Taken together, this evidence seems to indicate that as in external evaluation, evaluating one’s own creative product or that of someone else as deviant may inhibit that individual’s ability to generate creative products, thus reducing the number of ideas produced (*fluency*) and their quality (*originality*). Thus, a major purpose of the current study was to examine whether musical training in improvisation has a beneficial or “releasing” effect on creative abilities. Furthermore, we hypothesized that this effect results from training-related changes in the evaluation system, leading musicians trained in improvisation to see possible ideas as less deviant (see Fig. 2).

The present study examined whether the relationship between musical training, and specifically experience in improvisation, and the generation phase is mediated by the evaluation phase, thus lowering the evaluation inhibitory effect. To test this hypothesis we compared the performance of musicians with different types of musical training as well as of non-musicians on tasks that examined the generation and evaluation of creative products. We hypothesized that stricter evaluation would lead to lower scores on creativity. Therefore, based on previous findings showing higher divergent thinking scores among musicians, we hypothesized that musicians would have less strict evaluation. Furthermore, this study introduces a comparison never made before in the study of creativity, between musicians specializing in the performance of pre-composed music and musicians trained in improvisation. Nevertheless, this distinction is not clear-cut, since some individuals with musical training may have received more improvisation training than others. Furthermore, it may be argued that individual differences in creativity interact with the effects of training. Indeed, highly creative individuals may tend to choose to acquire training in a specific style of art. To rule out this possibility, we examined the amount of improvisation training and hypothesized that among musicians, self-report measures of musical training in improvisation would influence the evaluation phase, causing them to see ideas as less deviant, which in turn would have an overall effect on divergent thinking (originality and fluency on the AUT and TTCT, see below for additional details).

## Materials and Methods

### Participants

One hundred thirty-one young adults participated in the experiment (68 men, 63 women). Participants with musical training (total of 92) were recruited through advertisements placed at various musical programs in Israel (Haifa University, the Rimon School of Jazz and Contemporary Music, Tel Aviv University, and the Hebrew University of Jerusalem). The non-musicians (total of 39) responded to advertisements placed at Haifa University. Following screening, 18 participants were excluded (16 musicians, two non-musicians) from the analysis due to reports of psychiatric or neurologic problems. The participants were between 18–39 years of age (M = 24.65, SD = 3.28), all right handed, and all Hebrew speaking. The musicians included individuals who had begun playing an instrument between the ages of 5 and 16 (M = 8.37, SD = 3.04) with an average of 15.7 (SD = 3.73) years in playing a harmonic/melodic instrument as well as vocalists who practice daily. Some of the musicians were self-taught and therefore the range of formal training was between 3 and 23 years (M = 11.13, SD = 4.10). The musicians were divided into an improvisation group (n = 36) and a non-improvisation group (n = 40) based upon a discriminative questionnaire about experience in improvisation. The questions included the age at which they started to improvise, number of years of formal/informal practice of improvisation, number of weekly training hours, and other questions about musical training and performance preferences. The musicians also reported the styles in which they perform and train. The general distribution showed that almost half reported playing classical music, while the rest reported playing other styles (e.g., jazz, rock, pop). In the non-improvisation group all the participants reported playing classical music as well as other styles. The improvisation group was much more diverse in the styles they played, with more than a quarter reporting playing jazz (The Distribution charts are shown in Figures S1, S2, S3 in the Supporting Information available online). Twenty-one participants from the non-improvisation group reported having some experience in improvisation, but were still included in the non-improvisation group because of their lack of interest and time invested in improvisation (M = 1.69, SD = 1.49; weekly hours invested in improvisation [WHI]) compared to those in the group of improvising musicians (M = 8.41, SD = 8.34). Informed signed consent was obtained from each participant in accordance with the human subjects’ research protocol approved by the University of Haifa Ethics Committee.

### Materials

The *Shipley Institute of Living Scale* was used to assess participants’ intellectual abilities [39], [40]. The Shipley Institute of Living Scale is a two-part self-administered test consisting of a verbal section and an abstraction section, comparable to the verbal and performance dichotomy of the Wechsler Adult Intelligence Scale (WAIS). The verbal section consists of 40 multiple-choice questions on which the respondent is asked to choose which of four given words is closest in meaning to a target word. The abstraction section consists of 20 questions that include sequences of numbers, letters, or words, with the final item in each sequence omitted. The respondent is required to complete each of the sequences [40]. The test was used to examine a possible confounding correlation between IQ and musical training [41], and between IQ and creativity tests (see [3]).

#### The Alternate Uses Task (AUT) [42]

Participants were shown a list of five common objects (shoe, button, stapler, drinking glass, cardboard box) and were asked to list as many alternative uses as possible for each object within a period of 10 minutes, while trying to think of original uses (the most common everyday use was indicated in parenthesis). Since there are no guidelines for the scoring of original responses on the AUT, original responses were defined as statistically infrequent responses according to a previously conducted pretest (*N* = 65, [19]). The sample was later extended to include responses from 110 participants. An idea is awarded two points (2) if less than 3% of the participants in the pretest thought of the idea he or she proposed. One point (1) is awarded for ideas thought of by 3%–5% of pretest participants, and no points (0) are awarded for ideas thought of by more than 5% of pretest participants. Accordingly, originality is measured by the average accumulated points awarded for each item. Fluency is measured by the average of the number of items produced for each item. Flexibility is measured by dividing the number of categories produced by the number of items, thus producing a measure of the chance of producing an item in a new category. The categories were established using the categories given in the TTCT. The manuscript contains 68 categories (Torrance, 1974); if an item did not fall in any of them, a new category was created for that specific item.

#### The Evaluation Task

In the evaluation task participants were presented with 45 responses provided by other participants who had previously taken the AUT (ten different items). The responses were pre-divided into two levels of originality: high (2) and low (0). The responses were randomly selected from the bank of responses. The only selection criterion was that the originality value of a response was either high (2) or low (0). (For explanation of the division method, see above explanation of the AUT.) As mentioned above, the Alternative Uses Task included another level of originality (1) that was not included in the evaluation task. Participants were instructed to evaluate the deviance (D) by rating each item on a five-point rating scale, ranging from “not at all deviant” to “highly deviant”). Accordingly, reference to the deviance rating of a highly original item was indicated by “D2”.

#### Subset of Torrance Tests (circles) (TTCT, [43], [44])

Participants were instructed to draw as many drawings/ideas as they could from a matrix of circles. According to the instructions each drawing must be as unique as possible and must be assigned a title. Scoring of this test included the number of responses (fluency), the number of categories (flexibility), and the average statistical infrequency of the responses among a group of peers (originality). Unlike the AUT, the TTCT has a validated scoring system and was therefore scored according to the published manual. The flexibility measure used was similar to the one we used in the Alternative Uses task.

### Procedure

The above tasks were administered to each participant separately. Before administration of the tasks, participants completed a questionnaire of demographic questions (age, sex, and years of academic education) and the musical questionnaire mentioned above. After the participant signed the informed consent form, the tasks were administered in random order, except for the Evaluation Task, which was administered after the Alternative Uses Task (AUT). The overall duration of a single run was one hour.

## Results

MANOVA analysis revealed significant between-group differences in the participants’ age, years of academic education, and intelligence test scores. Therefore, these three factors were controlled throughout the analysis by adding them as covariates in order to negate alternative explanations of our results related to these factors. We divided the participants into three groups based on self-reported measures of musical activity, preferences, and training. T-test analyses showed that the groups of musicians did indeed differ in emphasis placed on improvisation training (see Table 1). While they did not differ in age at which they started their musical training, significant differences were found in the number of years of formal training (see Table 1).

**Table 1:** Self-report Measures of Musical Training among Musicians with and without Improvisation Training.

	With ImprovisationTraining (n = 36)		WithoutImprovisationTraining (n = 40)		Cohen’s d	p
Variables	M	SD	M	SD		
Age at start of musical training	8.97	3.13	8.05	3.12	.29	.809
Years of formal training	9.58	4.26	12.30	3.77	.68	.029
Age at start of improvisation training	14.92	4.34	15.80	5.43	.18	.542
Years of improvisation training	10.38	5.47	3.25	5.29	1.34	<.000
Weekly hours invested in improvisation	8.41	8.34	0.87	1.19	1.32	<.000
Weekly hours invested in performancewithout improvisation	6.40	5.78	17.29	14.92	−0.96	<.000
Weekly hours invested in composition	3.96	4.31	.074	2.05	1.19	<.000
Relative time invested in improvisation	0.41	0.24	0.07	0.13	1.81	<.000
Relative time invested in performancewithout improvisation	0.35	0.22	0.88	0.20	−2.56	<.000
Relative time invested in composition	0.25	0.22	0.05	0.15	1.09	<.000

### Between-Group Differences in Creativity

**Figure 3:**
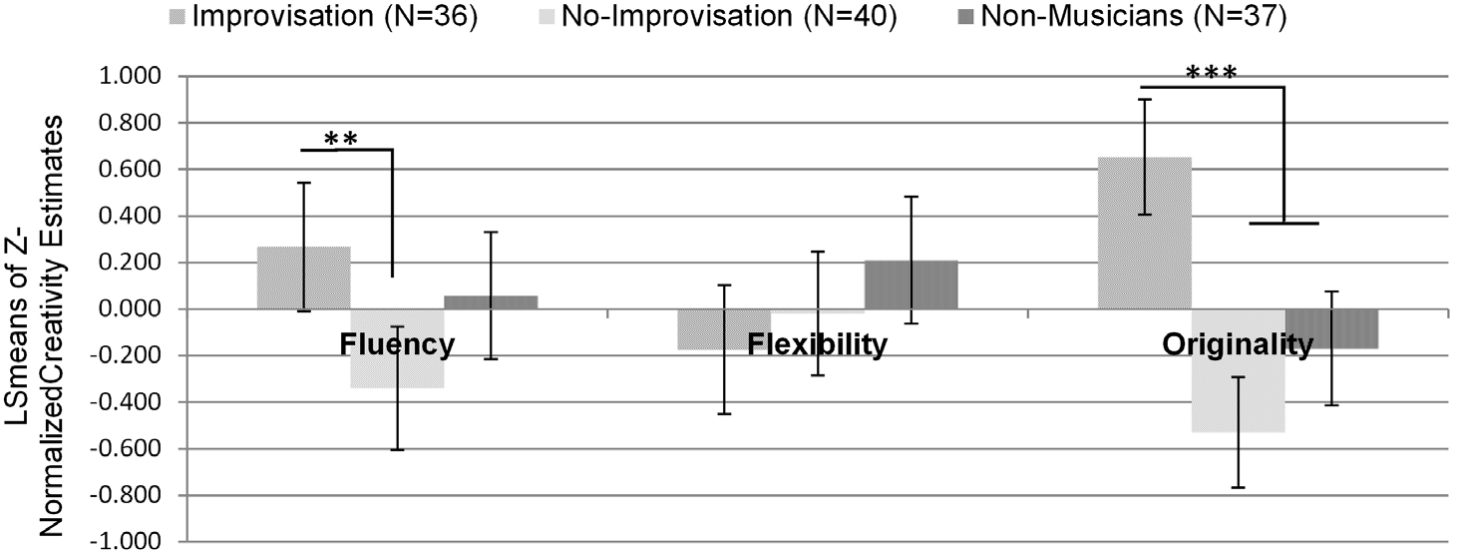
Between-group differences on the combined divergent thinking tasks (AUT, circles task). Model Least-squares means (LS means) that are generalizations of covariate-adjusted means were used with simulation-based 95% CI; Control variables: age, years of academic education and participants’ intelligence level; *p<0.05, **p<0.01, ***p<0.001.

The Alternative Uses Task (AUT) and the circles task from the TTCT were used to evaluate between-group differences in creativity. The fluency/flexibility/originality scores on these two tasks were combined by averaging their Z-scores, as described in Shamay-Tsoory et al. [19]. In line with our original hypothesis, between-groups differences were found (see Fig. 3). MANOVA testing was conducted on the three indices of creativity and the three groups. The Wilks’ Lambda correction revealed a significant influence of group on creativity [F(6,202) = 12.46, p<0.001, partial η^2^ = 0.27]. Follow-up univariate tests revealed that this influence originated from the significant differences between the groups in fluency [F(2,103) = 4.82, p = 0.01, partial η^2^ = 0.09] and in originality [F(2,103) = 23.55, p<0.001, partial η^2^ = 0.31]. However, no group differences emerged in flexibility [F(2,103) = 1.85, n.s]. Additional post hoc analyses, adjusted for multiple testing using a simulation procedure based on Edwards and Berry [45], were conducted. These analyses revealed that the improvisation group had higher fluency scores than the non-improvisation group (LSmean difference = 0.60, CI 95% [0.13, 1.08], p = 0.009), while no other group differences were found. The originality scores showed a similar trend, revealing that the improvisation group had higher originality scores than the non-improvisation group (LSmean difference = 1.18, CI 95% [0.76, 1.60], p<0.001) and the non-musicians group (LSmean difference = 0.82, CI 95% [0.39, 1.25], p<0.001), while no significant difference was found between the non-improvisation group and the non-musicians group.

### Evaluation Task

**Figure 4:**
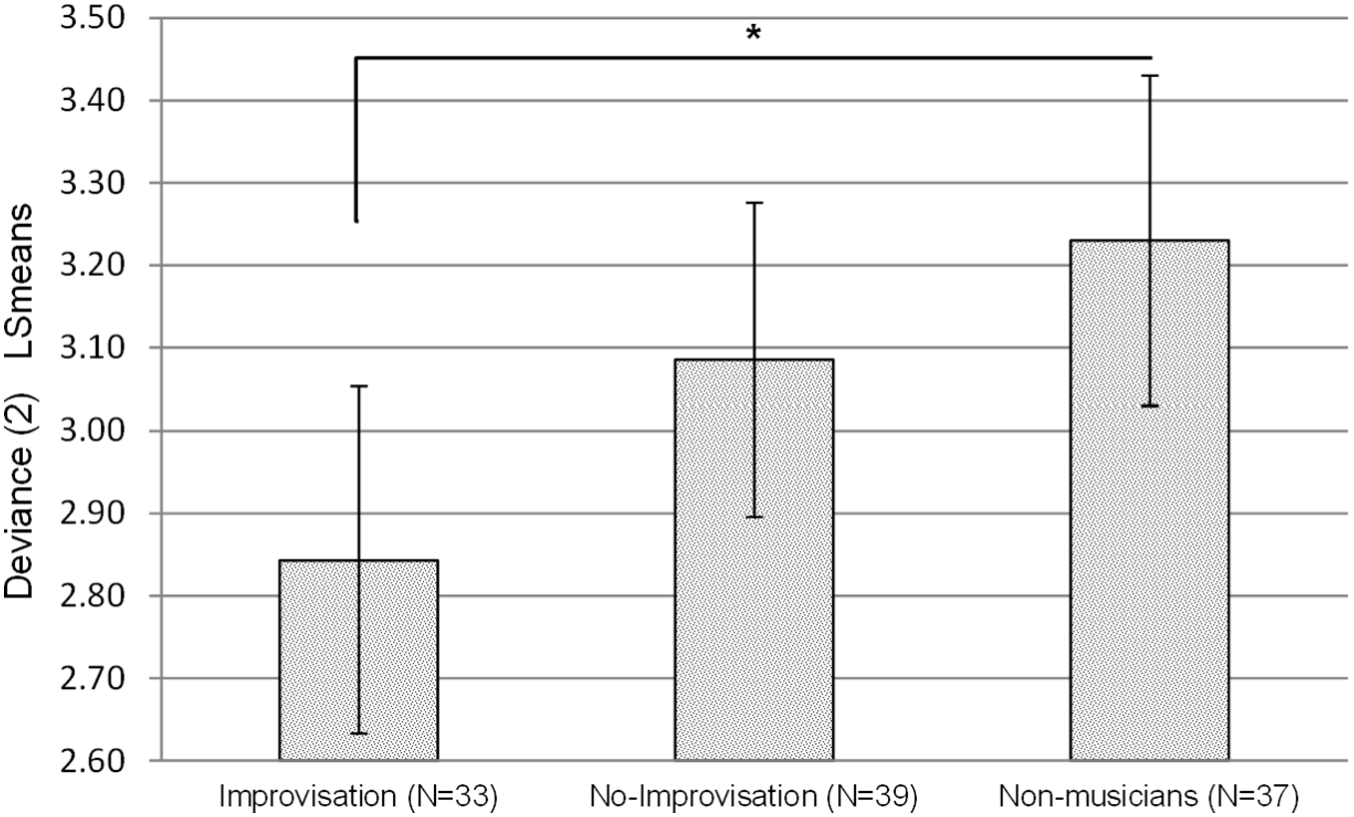
Between-group differences in deviance ratings of the most original items (2). Least-squares means (LS means) are generalizations of covariate-adjusted means used with simulation-based 95% CI; Control variables: age, years of academic education, and participants’ intelligence level; *p<0.05.

MANOVA analysis revealed significant group differences in deviance ratings [F(4,202) = 4.07, p = 0.003, partial η^2^ = 0.08]. Univariate analysis revealed group differences for D2 [F(2,102) = 6.49, p = 0.002, partial η^2^ = 0.11], but no group differences for D0 [F(2,102) = 1.75, n.s]. Post hoc analyses adjusted for multiple testing using a simulation procedure based on Edwards and Berry [45] revealed that the improvisation group had lower deviance scores than the non-musicians (LSmean difference = −0.54, CI 95% [−0.92, −0.17], p = 0.002), and marginally lower deviance scores than the non-improvisation group (LSmean difference = −0.36, CI 95% [−0.74, −0.001], p = 0.05) (see Fig. 4).

### Mediation analysis

We tested the mediating role of the evaluation indices (D2 and D0) on the improvisation-creativity relationship; improvisation as measured by years of improvisation training [YIT] and weekly hours invested in improvisation [WHI]) on the one hand, and creativity measured by the three creativity indices on the other (see Fig. 2). Multiple mediation with bootstrapping [46], using the PROCESS software package [47] was employed to examine the role of D2 and D0 in the improvisation-creativity relationship. Separate analyses were conducted for each combination of creativity indices and improvisation experience. All analyses included a bias-corrected bootstrap 95% confidence interval (CI) based on 5,000 bootstrap samples. Evidence for mediation (or indirect effect) in the bootstrap samples is observed with the absence of zero in the 95% bias corrected and accelerated confidence interval (Bca CI; [46]). The bootstrapping procedure is considered to be superior to conventional mediation models because it does not assume that the population is normally distributed, thus reducing Type I error rates [46].

Table 2 depicts the results of mediation analysis for WHI. As in previous analyses, all mediation models were controlled for age, years of academic education, and participants’ intelligence level. WHI emerged as a significant and negative predictor of D2 (b = −.03, SE = .01, p = .035), but not of D0 (b = −.01, SE = .01). Additionally, D2 was negatively related to fluency and originality, conditioned on the effect of WHI (b = −.51, SE = .20, p = .014) and (b = −.55, SE = .22, p = .014 respectively) (full model information can be found in supporting information, Table S1). However, D2 did not predicted flexibility conditioned on the effect of WHI (b = .19, SE = .19, n.s).

**Table 2:** The Mediating Effect of Evaluation on the Relationship between Improvisation Experience and Creativity.

The Mediation Path	X–M a path	M(X)–Y b path	X(M)-Y c’ path	The MediationEffect	S.E	Bootstrapping (95%) CI	
						Lower Limit	Upper Limit
WHI→D2→fluency	−.03*	−.51*	−0.01	.015*	0.009	0.001	0.038
WHI→D0→fluency	−0.01	−0.05	−0.01	<.001	0.003	−0.005	0.009
WHI→D2→originality	−.03*	−.55*	0.02	.016*	.010	0.001	0.044
WHI→D0→originality	−0.01	−0.07	0.02	<.001	0.003	−0.017	0.005
WHI→D2→flexibility	−.03*	0.19	0.01	0.006	0.006	−0.022	0.006
WHI→D0→flexibility	−0.01	0.24	0.01	0.002	0.004	−0.017	0.002

Note: *p≤0.05; Bootstrap sample size = 5000; CI  =  Confidence Interval; X - Weekly Hours invested in Improvisation (WHI), M – Deviance, Y – creativity induces.

Results from Hayes’s [47] bootstrapping method provided further support for the existence of a mediation effect of D2 - fluency: indirect effect = .015, SE  = .01, 95% confidence interval (CI) [.001, .038]; originality: indirect effect = .016, SE  = .01, 95% CI [.001, .044]. Nevertheless, the direct effect of WHI on fluency, originality and flexibility was not significant (b = −.01, SE = .02, n.s; b = .02, SE = .02, n.s; and b = .01, SE = .02, n.s, respectively). Additionally, YIT was not related either to D2 or to D0 (b = .02, SE = .02, n.s; and b = .01, SE = .01, n.s, respectively) (full mediation analysis can be found in supporting information, Table S2). Thus, a nonzero mediating effect of D2 was discovered for the relationship between WHI with fluency and originality but not with flexibility. For example, according to the mediating effect, an additional hour spent in improvisation training was related to lower ratings of D2 items, which in turn was related to higher creativity scores.

## Discussion

The findings of this study suggest that musicians who are trained in improvisation are more creative than either musicians without improvisation training or non-musicians, as characterized by performance on divergent thinking tasks (originality and fluency indices). These noticeable between-group differences in divergent thinking are potentially explained by differences in the way musicians and non-musicians evaluate the products of creative thinking (deviance ratings). The data suggest that non-musicians assign higher ratings to deviance than do musicians with improvisation training. Hence, these findings imply that stricter evaluation may potentially lead to lower scores on divergent thinking tests. Furthermore, consistent with our model, among musicians evaluation seems to mediate the relationship between weekly time invested in improvisation, fluency, and originality. These results may have important implications regarding the amount of time musicians invest in a specific type of practice, and more generally regarding the different emphasis given during their training. Interestingly, the mediation effect was found specifically for weekly hours invested in improvisation, and not for the overall number of years of improvisation training, and the results suggest that the effect is also unrelated to the number of years of formal training (which was even slightly higher in the non-improvisation group). Nevertheless, more research is needed to further establish the causality and directionality of this relationship. As humans, our first behaviors are improvisational in nature. Through learning we may also put more and more restrictions and inhibitions on different aspects of our lives [48]. According to this idea, it is possible that musicians who do not practice improvisation actually practice/train not to improvise and or to be creative in an improvisatory way. These non-improvisers try to apply practice, knowledge and inhibition, whereas improvisers try to forsake these practiced known habits after they were properly learned [48].

### Limitations

As noted in the introduction, individual differences in creativity may be the basis of some between-group differences. Personality [49] and genetics [50] may direct people who have certain predispositions toward pursuing musical training that includes emphasis on improvisatory abilities. Nonetheless, the correlations among weekly hours invested in improvisation, creative thinking scores, and evaluation of creativity may suggest that training has a unique effect on creativity.

The fact that the improvisation group was found to have higher scores on the creative indices may not necessarily make them better artists or predict a brighter creative future. As shown by Getzels and Csikszentmihalyi [51], divergent thinking indices were not found to correlate with future success as an artist. According to this longitudinal study, in order to predict an artist’s success researchers should place more emphasis on problem-finding rather than problem-solving measures. Accordingly, researchers should look for exploratory behaviors before, during, and after the solving of the problem presented. Exploratory behaviors are actions taken by a person who is about to behave in a creative manner, or is already behaving in such a way, in order to find a creatively significant problem. These actions delay the problem-solving process and prevent premature fixation on an unoriginal problem that might lead to unoriginal solutions [51]. Early fixation on a melodic line or other musical decisions might influence the subsequent outcome. It would be interesting to examine whether these exploratory behaviors are linked to the measures of evaluation we presented here. Would stricter evaluation be linked with more exploratory behavior, or the opposite?

Another known predicament in creativity research is the use of divergent thinking tasks [52]–[54]. When trying to link divergent thinking tasks and improvisation, one must keep in mind that divergent thinking tasks are individualistic measures, while most improvisation practice is conversational and takes place in a group setting [52]. Thus, the similarity between the two is partial, and more group tasks of divergent thinking and improvisation are needed in order to get a bigger picture. We do think that adding a more subjective aspect to the evaluation task, namely having participants rate their own ideas, might be interesting and enriching (see [54] for examples).

Lastly, we wish to emphasize that deviance perception is not a universal measure of creativity. Different cultures may have different definitions of creativity, and may consider different products as creative [48]. Although deviance is not a universal measure of creativity, cultural studies address the issue of deviance as affecting behavior [35]. Considering that enculturation has analogous characteristics to long periods of musical training from early childhood [55], [56], we believe that deviance perception may have analogous behavioral effects on musicians.

### Conclusion

Notwithstanding possible limitations, the results compel us to consider the importance of this line of study, and lead us to further elaborate on research examining the relation among type of training, creative thinking, and evaluation of creativity. Artists have been studied in the past in the context of creativity, and were found to score higher on creativity tests. Rarely, however, was their specific training put under the microscope in search of the practices that contribute to this advantage. Moreover, the contribution of evaluation of creativity is usually taken into account in sociocultural approaches to creativity research [3]. Our results suggest it might be interesting to consider its contribution at the level of the individual as well, which also takes into account sociocultural influences such as type of training and expertise. Finally, the implications of these findings should be considered in professional as well as educational settings to see how students can be taught to evaluate without compromising their creative products.

## Supporting Information

Figure S1 **General Distribution of Style.**
(TIF)

Figure S2 **The Improvisation Group: Distribution of Style.**
(TIF)

Figure S3 **The Non-Improvisation Group: Distribution of Style.**
(TIF)

Table S1 Full Mediation Model Information for WHI.
(DOCX)

Table S2 Full Mediation Model Information for YIT.
(DOCX)
