# Supplementary figures and images for: Expertise in Musical Improvisation and Creativity: The Mediation of Idea Evaluation
Source: PLoS One. 2014 Jul 10;9(7):e101568. doi: 10.1371/journal.pone.0101568 (PMC4092035; doi:10.1371/journal.pone.0101568)

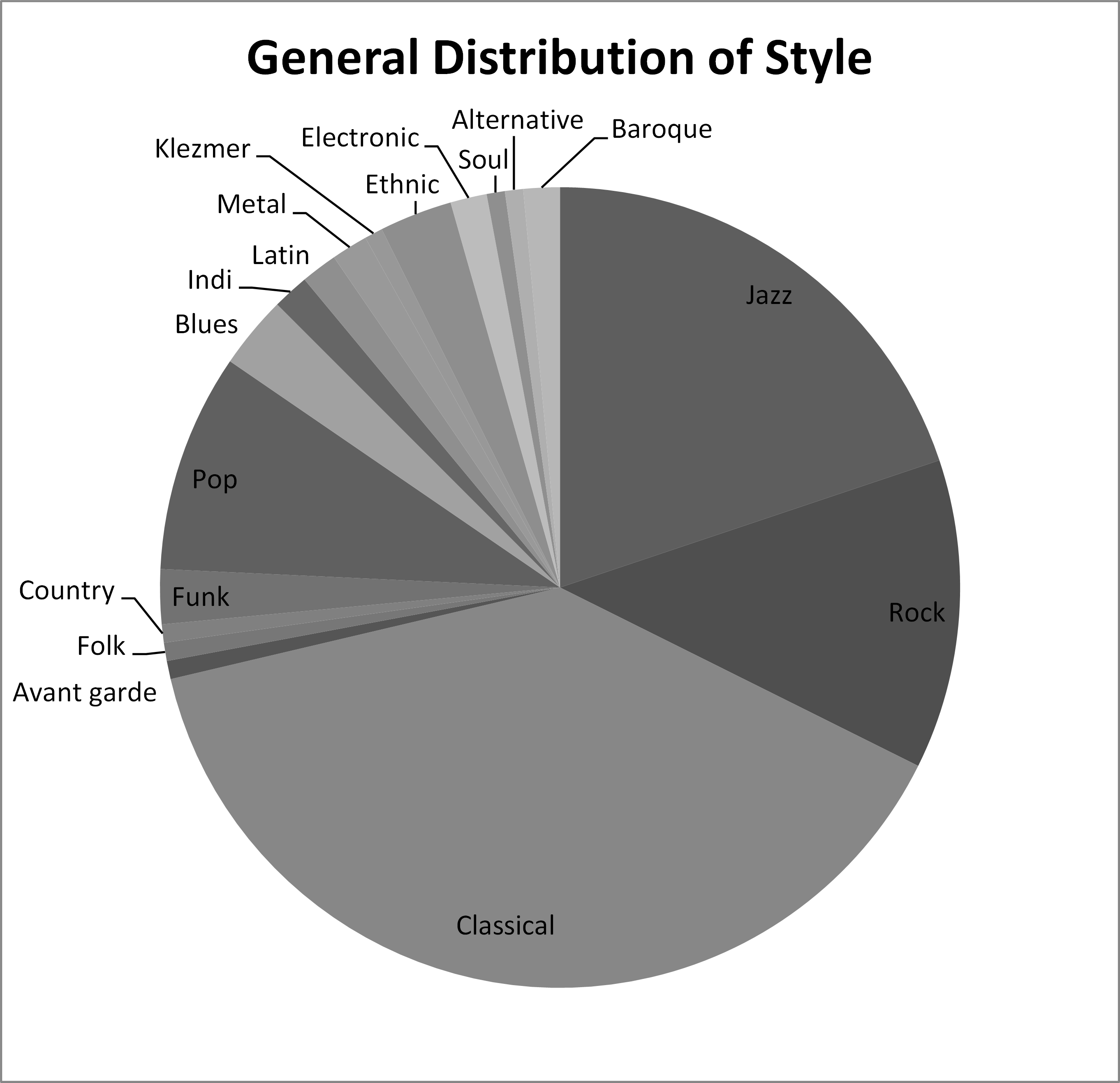

Supplement: Figure S1 — General Distribution of Style. (TIF) [file pone.0101568.s001.tif]

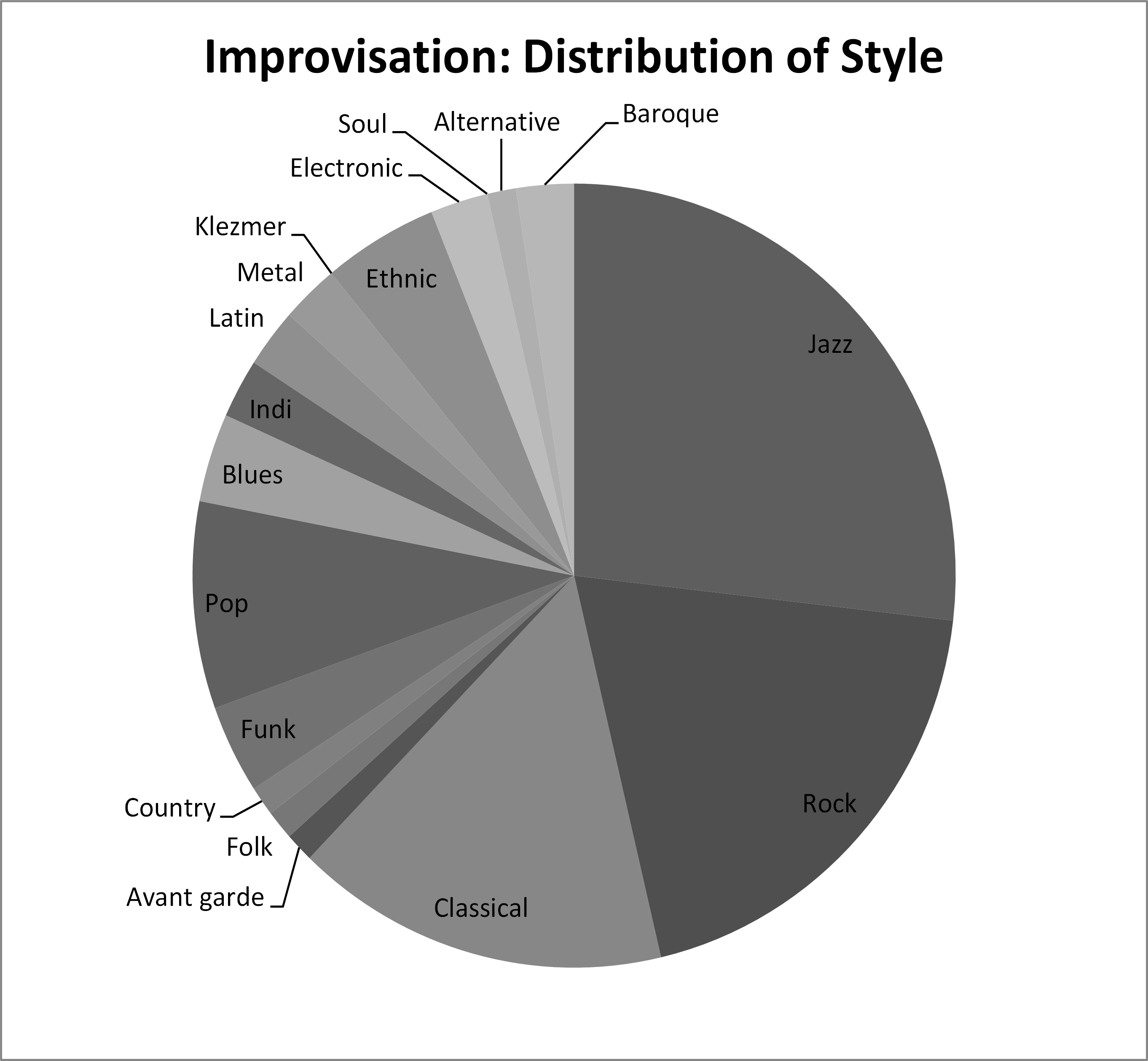

Supplement: Figure S2 — The Improvisation Group: Distribution of Style. (TIF) [file pone.0101568.s002.tif]

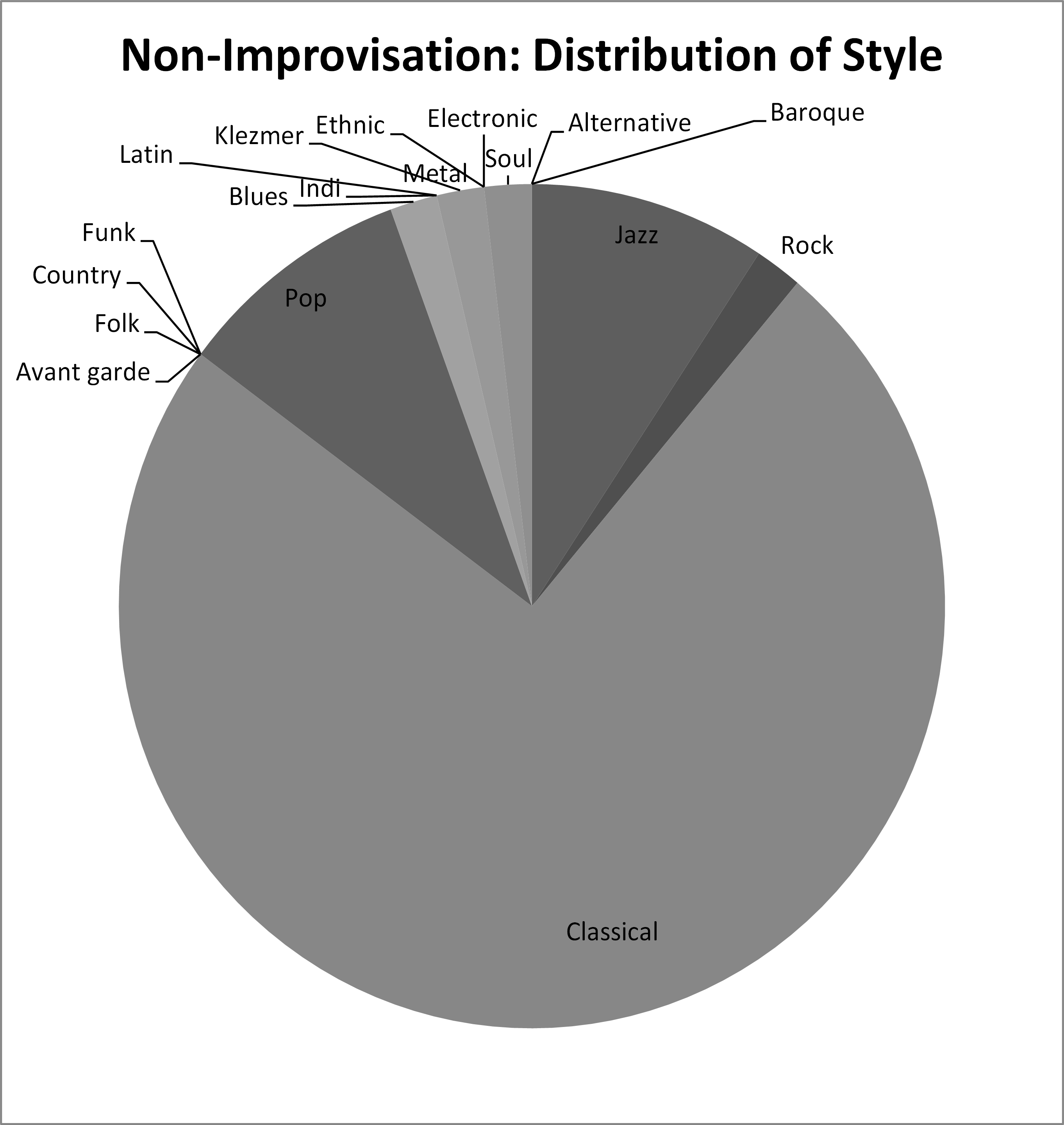

Supplement: Figure S3 — The Non-Improvisation Group: Distribution of Style. (TIF) [file pone.0101568.s003.tif]
