# Supplementary material for: Expertise in Musical Improvisation and Creativity: The Mediation of Idea Evaluation
Source: PLoS One. 2014 Jul 10;9(7):e101568. doi: 10.1371/journal.pone.0101568 (PMC4092035; doi:10.1371/journal.pone.0101568)
Supplement: Table S1 — Full Mediation Model Information for WHI. (DOCX) [file pone.0101568.s004.docx]

**Supplementary 1**

| outcome: D2 | B | SE | T | CI 95% | |
| --- | --- | --- | --- | --- | --- |
| *WHI* | *-0.03* | *0.01* | *-2.37** | *-0.05* | *-0.01* |
| age | -0.01 | 0.03 | -0.65 | -0.06 | 0.03 |
| education | -0.01 | 0.04 | -0.14 | -0.09 | 0.08 |
| Verbal intelligence | -0.02 | 0.01 | -1.93 | -0.04 | 0.01 |
| Performance intelligence | 0.03 | 0.03 | 1.07 | -0.03 | 0.09 |

Model 1: Relationship between Improvisation Experience and D2 conditioned on control variables (path a)

*p<.05

Model 2: Relationship between D2 and Originality conditioned on control variables and Improvisation Experience (path b)

| outcome: originality | B | SE | T | CI 95% | |
| --- | --- | --- | --- | --- | --- |
| *D2* | *-0.55* | *0.23* | *-2.39** | *-0.91* | *-0.09* |
| D0 | -0.05 | 0.43 | -0.13 | -0.91 | 0.80 |
| WHI | 0.02 | 0.02 | 1.14 | -0.02 | 0.07 |
| age | 0.01 | 0.04 | 0.13 | -0.07 | 0.08 |
| education | 0.12 | 0.07 | 1.76 | -0.02 | 0.26 |
| Verbal intelligence | 0.01 | 0.02 | 0.39 | -0.03 | 0.05 |
| Performance intelligence | 0.02 | 0.05 | 0.34 | -0.08 | 0.11 |

*p<.05

Model 3: Relationship between D2 and Fluency conditioned on control variables and Improvisation Experience (path b)

| outcome: fluency | B | SE | T | CI 95% | |
| --- | --- | --- | --- | --- | --- |
| *D2* | *-0.51* | *0.21* | *-2.43** | *-0.91* | *-0.09* |
| D0 | -0.05 | 0.06 | 0.92 | -0.19 | 0.07 |
| WHI | 0.01 | 0.02 | 0.45 | -0.03 | 0.05 |
| age | -0.01 | 0.03 | -0.37 | -0.08 | 0.05 |
| education | 0.03 | 0.06 | 0.56 | -0.09 | 0.16 |
| Verbal intelligence | -0.02 | 0.02 | -1.02 | -0.05 | 0.02 |
| Performance intelligence | 0.04 | 0.04 | 0.84 | -0.05 | 0.12 |

*p<.05
