# Supplementary material for: Expertise in Musical Improvisation and Creativity: The Mediation of Idea Evaluation
Source: PLoS One. 2014 Jul 10;9(7):e101568. doi: 10.1371/journal.pone.0101568 (PMC4092035; doi:10.1371/journal.pone.0101568)
Supplement: Table S2 — Full Mediation Model Information for YIT. (DOCX) [file pone.0101568.s005.docx]

**Table 2.** Mediating effect of evaluation on the relationship between years of improvisation training (YIT ) and creativity.

| Bootstrapping  (95%) CI | | S.E | The Mediation Effect | X(M) - Y  c' path | X) – Y)M  b path | X – M  a path | The Mediation Path |
| --- | --- | --- | --- | --- | --- | --- | --- |
| Upper  Limit | Lower  Limit |  |  |  |  |  |  |
| .020 | -.001 | .004 | .004 | .01 | -.26 | -.01 | YIT → D2 → fluency |
| .008 | -.009 | .004 | <.001 | .01 | -.48* | <.01 | YIT→ D0 → fluency |
| .020 | -.001 | .010 | .010 | .04 | -.25 | -.01 | YIT→ D2 → originality |
| .008 | -.007 | .004 | <.001 | .04 | -.33 | <.01 | YIT→ D0 → originality |
| .001 | -.010 | .003 | -.003 | .01 | .20 | -.01 | YIT→ D2 → flexibility |
| .007 | -.008 | .004 | <.001 | .01 | .40 | <.01 | YIT→ D0 → flexibility |

Note: *p ≤ 0.05; Bootstrap sample size = 5000; CI = Confidence Interval; X - Years of Improvisation Training (YIT), M – Deviance, Y – creativity indices.
